# Glutamate receptor-mediated taurine release from the hippocampus during oxidative stress

**DOI:** 10.1186/1423-0127-17-S1-S10

**Published:** 2010-08-24

**Authors:** Brian Tucker, James E Olson

**Affiliations:** 1 Department of Neuroscience, Cell Biology, and Physiology, Boonshoft School of Medicine, Wright State University, Dayton, Ohio, USA; 2 Department of Emergency Medicine, Boonshoft School of Medicine, Wright State University, Dayton, Ohio, USA

## Abstract

**Background:**

Hippocampal slices swell and release taurine during oxidative stress. The influence of cellular signalling pathways on this process is unclear. Glutamate signalling can facilitate volume regulation in other CNS preparations. Therefore, we hypothesize activation of taurine release by oxidative stress results from tissue swelling and is coupled to activation of glutamate receptors.

**Methods:**

Rat hippocampi were incubated at room temperature for 2 hr in artificial cerebrospinal fluid (aCSF) equilibrated with 95% O_2_ plus 5% CO_2_.  For some slices, 1 mM taurine was added to the aCSF to maintain normal tissue taurine content. Slices then were perfused with aCSF at 35° C and baseline data recorded before 2 mM H_2_O_2_ was added. For some studies, mannitol or inhibitors of glutamate receptors or the volume-regulated anion channel (VRAC) were added before and during H_2_O_2_ treatment. The intensity of light transmitted through the slice (the intrinsic optical signal, IOS) was determined at 1-min intervals. Samples of perfusate were collected at 2-min intervals and amino acid contents determined by HPLC. Data were analyzed by repeated measures ANOVA and *post hoc* Dunnett’s test with significance indicated for p<0.05.

**Results:**

IOS of slices prepared without taurine treatment increased significantly by 3.3±1.3% (mean±SEM) during oxidative stress. Little taurine was detected in the perfusate of these slices and the rate of taurine efflux did not change during H_2_O_2_ exposure. The α-amino-3-hydroxyl-5-methyl-4-isoxazole-propionate antagonist, 25 µM CNQX, but not the N-methyl-D-aspartate (NMDA) receptor antagonist, 10 µM MK-801, inhibited the increase in IOS during H_2_O_2_ treatment. Taurine-treated slices exposed to H_2_O_2_ showed no change in IOS; however, taurine efflux increased by 335±178%. When these slices were perfused with hypertonic aCSF (350 mOsm) or exposed to the VRAC inhibitor, 20 µM DCPIB, no increase in the taurine efflux rate was observed during H_2_O_2_ exposure. Taurine-treated slices perfused with 10 µM MK-801 during H_2_O_2_ exposure showed a 4.6±1.9% increase in IOS but no increase in the taurine efflux rate.

**Conclusions:**

Taurine efflux via VRAC is critical for volume regulation of hippocampal slices exposed to oxidative stress. This increased taurine efflux does not result from direct activation of the taurine release pathway by H_2_O_2_. NMDA receptor activation plays an important role in taurine release during oxidative stress.

## Background

Oxidative stress is observed following brain injury caused by a variety of mechanisms and contributes to secondary brain injury. Brain edema in ischemic and hemorrhagic stroke is associated with production of reactive oxygen species (ROS) and resultant oxidative stress [1,2]. Endogenous and exogenous anti-oxidants reduce edema formation and brain damage following ischemia and reperfusion [1,3-5] and intracerebral hemorrhage [2,6]. Reactive oxygen species (ROS) may directly precipitate brain swelling without inducing ischemia or blood-brain barrier injury during intracranial hemorrhage and excitotoxic injury [7,8].

*In vitro s*tudies have shown oxidative stress can cause brain tissue swelling through activation of glutamate receptors [9]. Because of their direct activation of ion channels and resultant osmotic sequelae, ionotropic glutamate receptors may play a significant role in initiating this brain tissue swelling. Indeed, brain swelling during exposure to H_2_O_2_ is inhibited by ionotropic glutamate receptor antagonists [9]. Brain edema also is modified by metabotropic glutamate receptor activation in certain models of traumatic brain injury and excitotoxicity [10-12]; however, other investigators have seen no effect of the metabotropic group II receptor [12] on injury-induced brain edema.

In addition to causing brain edema, activation of glutamate receptors may modify the response of CNS cells to swelling through actions on cell volume regulatory mechanisms [13,14]. Neurons and glial cells, like many other cell types, facilitate swelling-induced volume regulation by activating volume-regulated anion channels (VRAC) which cause efflux of taurine and other organic osmolytes [15-17]. Thus, glutamatergic signaling during oxidative stress may indirectly lead to brain swelling by modifying this normal cell volume regulation mechanism. This study focuses on ionotropic glutamate signaling during hippocampal oxidative stress. We examine whether this pathway directly induces brain swelling or modifies brain cell volume regulation via the VRAC.

## Methods

All experiments involving animals were approved by the Laboratory Animal Care and Use Committee of Wright State University and conform to the Guide for the Care and Use of Laboratory Animals. Slices of hippocampus were prepared using methods similar to those previously described [18]. Adult Sprague Dawley male rats (275-350 g) were anesthetized to apnea with Isoflurane and then perfused over 1 min via the left cardiac ventricle with 100 ml of ice-cold artificial cerebrospinal fluid (aCSF) consisting of 124 mM NaCl, 3.5 mM KCl, 2 mM CaCl_2_, 1 mM MgSO_4_, 1 mM NaH_2_PO_4_, 26 mM NaHCO_3_, and 10 mM glucose. The aCSF osmolality was adjusted to 290 mOsm with additions of small volumes of 3 M NaCl and was equilibrated with 95% O_2_ plus 5% CO_2_ prior to use. This perfusion treatment flushed away blood and rapidly chilled the brain tissue. The brain was removed from the head and placed in a slurry of ice-cold aCSF. After 5-6 min, the hippocampus was dissected from the brain and cut into 400 µM slices with a McIlwain chopper. These slices were incubated for 2-4 hr in room temperature aCSF that was constantly bubbled with 95% O_2_ plus 5% CO_2_. Hippocampal slices prepared in this manner lose 70% to 80% of endogenous taurine contents [18]. Therefore, for some slices, the aCSF solutions used during incubation also contained 1 mM taurine to maintain normal taurine contents.

After the room temperature incubation period, slices were transferred to the recording stage of a Haas-type interface chamber and perfused with aCSF equilibrated with 95% O_2_ plus 5% CO_2_ at 35° C under an atmosphere of humidified 95% O_2_ plus 5% CO_2_. For slices exposed to 1 mM taurine during room temperature incubation, the same taurine concentration was present during the first 30 min of perfusion on the recording stage. After a total of 90 min on the recording stage, 2 mM H_2_O_2_ was added to the perfusing aCSF to produce oxidative stress. For slices treated with pharmacological agents, the drugs were added to the aCSF beginning 15 min prior to the start and then throughout the period of H_2_O_2_ exposure.

The intrinsic optical signal (IOS) was recorded as an indirect measure of changes in tissue volume [18,19]. Slices were illuminated with a DC regulated light source while the light transmitted through the slice was recorded with a video camera and captured as uncompressed digital images every 60 sec. The average pixel intensity was calculated for identified regions of the stratum radiatum of the CA1 region interest using ImageJ software.

Effluent perfusate from the recording stage was collected continually during the experiments and pooled in 2 min aliquots. A 1 ml sample was removed from each aliquot, frozen at -70° C, lyophilized, and then stored at -70° C for later analysis. The residue was dissolved in 100 µl of distilled water and 65 µl was used for HPLC analysis of amino acid concentration following derivatization with o-pthalaldehyde as previously described [18,20].

AMPA (α-amino-3-hydroxyl-5-methyl-4-isoxazole-propionate), CNQX (6-cyano-7-nitroquinoxaline-2,3-dione), and MK-801 ((+)-5-methyl-10,11-dihydro-5H-dibenzo[a,d]cyclohepten-5,10-imine maleate) were obtained from Sigma-Aldrich (St. Louis, Missouri). DCPIB (4-[(2-Butyl-6,7-dichloro-2-cyclopentyl-2,3-dihydro-1-ox o-1H-inden-5-yl)oxy]butanoic acid) was purchased from Tocris Biosciences (Ellisville, Missouri). All other chemicals were from Fischer Scientific (Hanover Park, Illinois) and were the highest grade available.

IOS and amino acid contents were analyzed by repeated measures ANOVA followed by *post hoc* Dunnett’s test to compare data obtained during oxidative stress with values measured during the baseline period prior to H_2_O_2_ exposure. Significance was indicated for p<0.05.

## Results

### Taurine is necessary for volume regulation during oxidative stress

**Figure 1:**
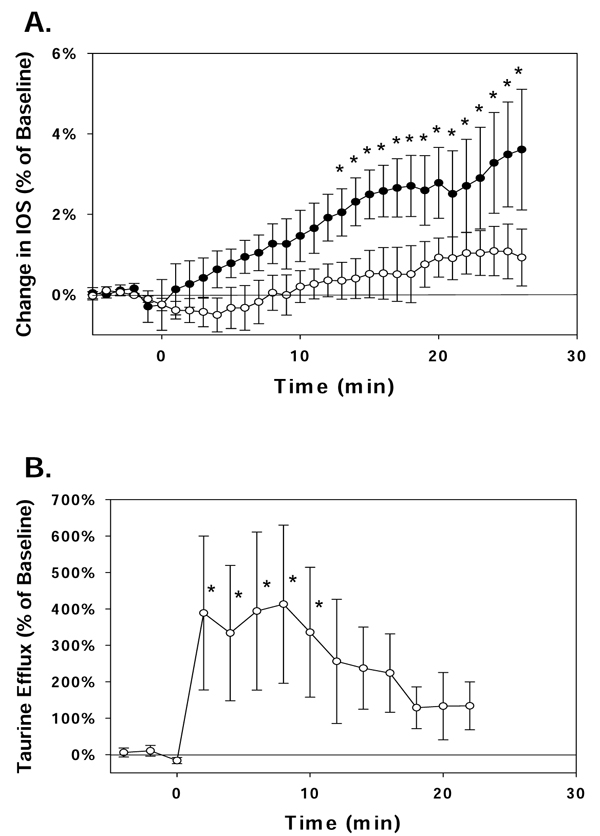
**Tissue swelling and taurine efflux from the hippocampus during oxidative stress.** Hippocampal slices were perfused with aCSF containing 2 mM H_2_O_2_ from t = 0 min to the end of the data sampling period shown. Values are the mean ± SEM for 3-12 independent measurements. (A) The intrinsic optical signal (IOS) was calculated for areas of the stratum radiatum in the CA1 region of the hippocampus. Slices incubated with normal aCSF during preparation (solid symbols) showed a significant increase in IOS during exposure to H_2_O_2_ while slices incubated with 1 mM taurine (hollow symbols) showed little change in IOS. (B) Taurine concentrations were measured in the effluent of the aCSF that perfused hippocampal slices prepared with 1 mM taurine to maintain normal taurine contents. The taurine concentration in the aCSF perfusate increased significantly at the start of H_2_O_2_ exposure and remained elevated for 10 min. * indicates values which are significantly different from the baseline measurements determined prior to H_2_O_2_ treatment.

Hippocampal slices prepared with incubation in normal aCSF without added taurine demonstrated a significant increase in IOS during exposure to H_2_O_2_ (Figure 1.A.). Little taurine was measured in the perfusate from these slices and the taurine concentration did not increase during peroxide exposure (data not shown). In contrast, slices incubated with 1 mM taurine showed little swelling throughout the period of oxidative stress while the concentration of taurine in the perfusate increased over 3-fold during this period (Figure 1.B.).

### Taurine efflux occurs via VRAC to control swelling during oxidative stress

We tested whether the increase in taurine release during oxidative stress was due to cell swelling or direct activation of VRAC by H_2_O_2_[21,22]. For these studies, the osmolality of the aCSF perfusing taurine-treated slices was increased to 350 mOsm with the addition of mannitol for 15 min prior to adding 2 mM H_2_O_2_ to the perfusate. Mannitol treatment markedly and rapidly decreased the IOS by 17.0±2.2% within 10 min (mean±SEM). No increase in taurine efflux was observed in these slices during the subsequent exposure to oxidative stress.

In the presence of the VRAC inhibitor, 20 µM DCPIB [23], slices incubated with taurine and then perfused with normal aCSF (290 mOsm) demonstrated an increase in IOS but no increase in taurine efflux during exposure to 2 mM H_2_O_2_ (Table 1).

**Table 1:** Drug effects on tissue swelling and taurine efflux from the hippocampus during oxidative stress.

Drug Treatment	H_2_O_2_-induced Change in IOS (Percent of Baseline)	H_2_O_2_-induced Change in Taurine Efflux Rate (Percent of Baseline)
None	0.7 ± 0.6 %	335 ± 178 % *
20 µM DCPIB	3.2 ± 1.0 % *	-11 ± 29 %
10 µM MK-801	4.6 ± 1.9 % *	-55 ± 13 %

All experiments were performed using hippocampal slices prepared with 1 mM taurine to maintain normal taurine contents. Changes in the intrinsic optical signal (IOS) measured in the stratum radiatum of the CA1 region and the taurine efflux rate were determined after 25 min and 8 min of H_2_O_2_ exposure, respectively. These represent time points which resulted in peak responses observed in control slices with no drug treatment as shown in Figure 1. Values are the mean ± SEM for 3-5 independent measurements. * Indicates values which are significantly different from baseline measurements determined prior to H_2_O_2_ treatment.

### Glutamate signalling is coupled to volume regulation during oxidative stress

To examine the role of glutamate signalling for oxidative stress-induced swelling and volume regulation, hippocampal slices were exposed to an inhibitor of either N-methyl-D-aspartate (NMDA) receptors (10 µM MK-801) or AMPA receptors (25 µM CNQX). In slices without taurine treatment, no change in IOS was observed when 2 mM H_2_O_2_ was added to the perfusate in the presence of CNQX. This suggests AMPA receptors contribute to the mechanisms of swelling induced by oxidative stress. In contrast, in the presence of MK-801, the mean±SEM IOS increased by 1.8±0.2% during 25 min of peroxide exposure; a value that is similar to that observed in the absence of drug (Figure 1). However, when taurine-treated slices were exposed to H_2_O_2_ in the presence of MK-801, a significant increase in IOS was seen, and the rate of taurine efflux was not altered (Table 1).

## Discussion

The results of these experiments illuminate several mechanisms for swelling and volume regulation of the hippocampus exposed to oxidative stress. First, the data demonstrate the importance of intracellular taurine for volume regulation in this condition. We previously demonstrated that hippocampal slices lose the majority of their taurine content during preparation and incubation unless exogenous taurine is added to the incubation solutions [18]. Our previous data also demonstrated that a normal tissue taurine content was critical for volume regulation of the hippocampus swollen by exposure to hypoosmotic conditions. The present results reveal a similar requirement of normal taurine content for volume regulation during exposure to oxidative stress in isoosmotic conditions. Furthermore, inhibition of oxidative stress-induced taurine efflux by the VRAC antagonist, DCPIB, and the tissue swelling which results in the presence of this drug, indicates that taurine efflux is necessary for the volume regulatory response. Others have demonstrated the release of amino acids through VRAC channels during exposure to hypoosmotic conditions and oxidative stress [24-26]. We characterized VRAC in rat astrocytes and neurons in primary culture during exposure to hypoosmotic conditions [16,27] and showed that taurine efflux via VRAC is necessary for hypoosmotic volume regulation in the hippocampus [18]. Inhibition of oxidative stress-induced taurine efflux by pre-treatment with hyperosmolar aCSF demonstrates that H_2_O_2_ is not acting directly on VRAC to induce taurine efflux as has been observed in cell culture preparations [21,28].

Our data also suggest that the AMPA subtype of ionotropic glutamate receptors contributes to hippocampal swelling induced by oxidative stress while NMDA receptors have little effect. Previous reports have shown that swelling of hippocampal slices exposed to H_2_O_2_ for 1-3 hours can be blocked using a cocktail of glutamate receptor inhibitors; however, individual contributions of NMDA and AMPA receptors for the swelling response were not elucidated [29]. We did not examine the effects of metabotropic glutamate receptors in the brain tissue swelling response. Potentially, activation of group I or group II receptors may indirectly exacerbate or reduce swelling, respectively, by modifying endogenous glutamate release and resulting stimulation of ionotropic receptors [10,11,30].

Finally, our results demonstrate that glutamate signaling via NMDA receptors plays an important role in volume regulation of the hippocampus during oxidative stress. Since oxidative stress-induced swelling itself is blocked by CNQX, we could not determine whether AMPA receptors also may be involved in the volume regulatory response. We and others have described several signaling pathways involved in cell volume regulation and associated amino acid efflux from brain cells. These include the calcium/calmodulin pathway [24,31,32], nucleotide receptors [16,26], arachidonate metabolism [22,33] and glutamate receptor activation [13,14]. The relationships between these pathways as well as the interactions between glia and neurons which facilitate brain volume regulation have not yet been fully described. Greater understanding of these mechanisms will lead to improved treatments for brain edema and will reduce the degree of permanent brain damage in patients who suffer brain injury.

## Conclusions

1. Release of taurine from intracellular stores is critical for isoosmotic volume regulation of the hippocampus exposed to oxidative stress.

2. Taurine is lost from hippocampal cells during oxidative stress via the volume activated anion channel (VRAC).

3. AMPA receptors, but not NMDA receptors are coupled to hippocampal swelling during oxidative stress.

4. Volume regulation of the hippocampus is regulated, in part, by glutamate signaling via NMDA receptors.

## List of abbreviations

aCSF, artificial cerebrospinal fluid; AMPA, α-amino-3-hydroxyl-5-methyl-4-isoxazole-propionate; CNQX, 6-cyano-7-nitroquinoxaline-2,3-dione; DCPIB, 4-[(2-Butyl-6,7-dichloro-2-cyclopentyl-2,3-dihydro-1-ox o-1H-inden-5-yl)oxy]butanoic acid; IOS, intrinsic optical signal; MK-801, (+)-5-methyl-10,11-dihydro-5H-dibenzo[a,d]cyclohepten-5,10-imine maleate; NMDA, N-methyl-D-aspartate; ROS, reactive oxygen species; VRAC, volume-regulated anion channel

## Competing interests

Neither of the authors has competing interests regarding this manuscript.

## Authors' contributions

Both authors contributed to the conception of the study and its design. BT carried out the majority of the experimental studies and the analytical and statistical analyses. JO drafted the manuscript, but both authors contributed to its completion. Both authors read and approved the final manuscript.
